# Mendelian randomization study reveals a causal relationship between serum iron status and coronary heart disease and related cardiovascular diseases

**DOI:** 10.3389/fcvm.2023.1152201

**Published:** 2023-06-13

**Authors:** Fenglan Liu, Yanfei Liu, Shihan Xu, Qing Wang, Fengqin Xu, Yue Liu

**Affiliations:** ^1^ The Second Department of Geriatrics, Xiyuan Hospital, China Academy of Chinese Medical Sciences, Beijing, China; ^2^ National Clinical Research Center for TCM Cardiology, Xiyuan Hospital, China Academy of Chinese Medical Sciences, Beijing, China; ^3^ School of Clinical Medicine, Guangdong Pharmaceutical University, Guangzhou, China

**Keywords:** iron status, cardiovascular disease, causal effect, genome-wide association studies, Mendelian randomization

## Abstract

**Background:**

Growing observational studies have shown that abnormal systemic iron status is associated with Coronary heart disease (CHD). However, these results from observational studies was not entirely consistent.It remains unclear whether this relationship represents causality.It is necessary to explore the causal relationship between iron status and CHD and related cardiovascular diseases (CVD).

**Objective:**

We aimed to investigate the potential casual relationship between serum iron status and CHD and related CVD using a two-sample Mendelian randomization (MR) approach.

**Methods:**

Genetic statistics for single nucleotide polymorphisms (SNPs) between four iron status parameters were identified in a large-scale genome-wide association study (GWAS) conducted by the Iron Status Genetics organization. Three independent single nucleotide polymorphisms (SNPs) (rs1800562, rs1799945, and rs855791) aligned with four iron status biomarkers were used as instrumental variables. CHD and related CVD genetic statistics We used publicly available summary-level GWAS data. Five different MR methods random effects inverse variance weighting (IVW), MR Egger, weighted median, weighted mode, and Wald ratio were used to explore the causal relationship between serum iron status and CHD and related CVD.

**Results:**

In the MR analysis, we found that the causal effect of serum iron (OR = 0.995, 95% CI = 0.992–0.998, *p* = 0.002) was negatively associated with the odds of coronary atherosclerosis (AS). Transferrin saturation (TS) (OR = 0.885, 95% CI = 0.797–0.982, *p* = 0.02) was negatively associated with the odds of Myocardial infarction (MI).

**Conclusion:**

This MR analysis provides evidence for a causal relationship between whole-body iron status and CHD development. Our study suggests that a high iron status may be associated with a reduced risk of developing CHD.

## 1. Introduction

Due to an aging population and declining fertility rates, cardiovascular disease mortality continues to rise and imposes a considerable economic and health burden on society (1). As research progresses, more and more studies show the correlation between systemic iron status and heart disease and related CVD (2).

CHD remains one of the major diseases threatening the health of the entire human population (3). The development of CHD involves many associated CVD. Among CVD, MI, a type of coronary heart disease, is a serious consequence of coronary heart disease. Hypertension (HP) is not only a CVD but also one of the risk factors for CHD, while heart failure (HF) is the end-stage disease of most CVD including CHD.

Iron, an essential mineral for maintaining homeostasis in the body, plays a key role in oxygen transport and utilization as well as in mitochondrial function (4). Iron deficiency (ID) is associated with morbidity and mortality in CHD, M I and HF (5–8). Studies have shown that ID is one of the most common complications of HF. Iron supplementation via intravenous can reduce the number of hospitalizations for HF (9). It has been shown that ID impairs the contractility of human cardiomyocytes by reducing mitochondrial function and decreasing energy production, which leads to impaired cardiac function (10). When uncontrolled elevation of iron concentration leads to iron overload, the basic cellular mechanisms and functional composition are disrupted and changed (11). The redox properties of iron enable the generation of reactive oxygen species (ROS), and iron (Fe^2+^) and iron (Fe^3+^) mediate lipid peroxidation, leading to the formation of alkoxyl (RO) and peroxyl (RO^2^) radicals (12, 13). Studies have displayed that in animal models of ischemia/reperfusion (I/R) cardiac tissue samples it can be observed that increased mitochondrial iron-related reactive oxygen species (ROS) production leads to myocardial injury (14). However, the physiopathological mechanisms of ID and iron overload participating in CHD and associated CVD remain unclear. In conclusion both opposite factors, ID and iron overload, can have an impact on CHD and related CVD. However, even in observational studies it is difficult to distinguish which specific association exists between iron status and CHD and associated CVD, as selective bias or other biases inherent in observational studies can still influence the results. Therefore, further studies are necessary to elucidate whether there is a causal or other relationship between iron status and CHD and associated CVD.

MR analysis is a novel method of epidemiological analysis that strengthens causal inferences by using genetic variation as an instrumental variable (IV) such as SNPs for exposure. This method minimizes the effects of residual confounding and strengthens causal inferences about the effects of specific exposure factors on outcomes while overcoming the limitations of traditional epidemiological studies (15). Here, we performed a 2-sample MR study to examine the association of iron status with HP, AS, CHD, MI, and HF based on the effect of systemic iron status and CHD and associated CVD using GWAS data, aiming to provide new evidence on the relationship between iron status and disease progression of CHD.

## 2. Materials and methods

### 2.1. Study design

**Figure 1:**
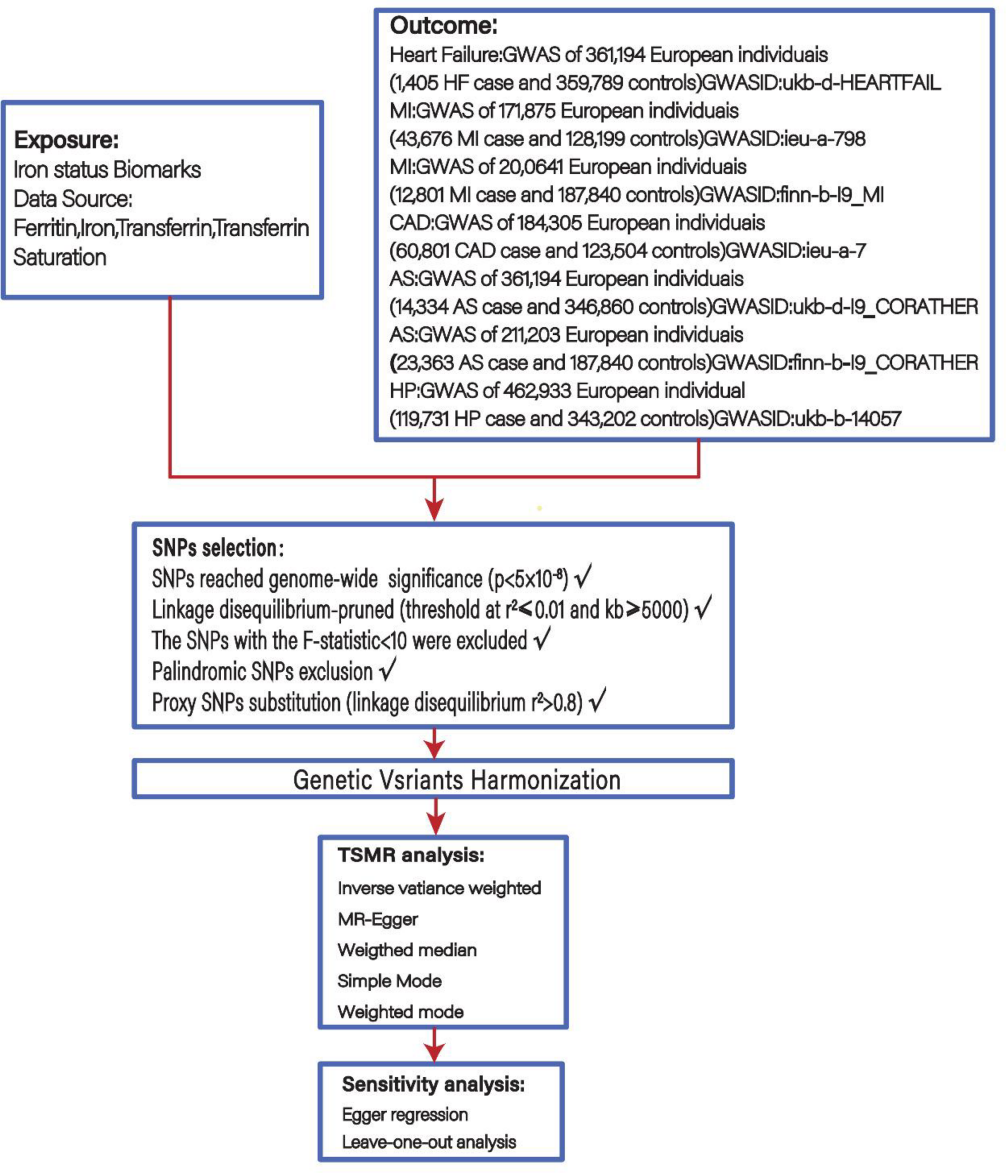
Flowsheet of Mendelian randomisation in this study.

A genetic tool of four iron status biomarkers: ferritin, iron, transferrin, and transferrin saturation (TS), was selected for a two-sample MR analysis as a way to investigate the association of iron status biomarkers with the chain of cardiovascular disease events including HP, AS, CHD, MI and HF. The screening flowsheet is shown in Figure 1.

### 2.2. Selection of instrumental SNPs

Genetic variants associated with serum iron status were identified through a meta-analysis of 19 GWAS, which included 48,972 Europeans (16). A higher level of systemic iron was associated with higher iron levels, higher transferrin saturation, and higher ferritin levels, but decreased transferrin levels (4, 16, 17). Thus, genetic tools for iron status should be consistently related to each of these four markers, and thus three loci (rs1800562 and rs1799945 in the HFE gene, and rs855791 in TMPRSS6) could be identified in the meta-analysis performed by the GIS Consortium as being significantly associated with all four iron status markers genome-wide (*p *< 5 × 10^−8^) in a pattern consistent with effects on systemic iron status (i.e., increased serum iron, transferrin saturation and ferritin levels and decreased transferrin levels) (16), and these three were suggested as tools for systemic iron status in our MR analysis. Three SNPs had *p *< 5 × 10^−8^ and *r*^2^ ≤ 0.01, and their F-statistics were calculated to quantify the intensity of the selected instrument (F-value > 10) (18).

Characteristics and summary data of the SNPs and iron status parameters shown in Table 1 and Supplementary Table S1, respectively.

**Table 1:** The characteristics and summary data of the exposed SNPs and iron status parameters.

	SNP	Nearest gene	Effect allele	Other allele	Eaf	F	Beta	SE	*P*
Iron	rs1799945	HFE (H63D)	C	G	0.85	450	−0.189	0.01	1.10 × 10^−81^
	rs1800562	HFE (C282Y)	A	G	0.067	668	0.328	0.016	2.72 × 10^−97^
	rs855791	TMPRSS6 (V736A)	A	G	0.446	806	−0.181	0.007	1.32 × 10^−139^
Ferritin	rs1799945	HFE (H63D)	C	G	0.85	52	−0.065	0.01	1.71 × 10^−10^
	rs1800562	HFE (C282Y)	A	G	0.067	256	0.204	0.016	1.54 × 10^−38^
	rs855791	TMPRSS6 (V736A)	A	G	0.446	73	−0.055	0.007	1.38 × 10^−14^
Transferrin	rs1799945	HFE (H63D)	C	G	0.85	676	0.114	0.01	9.36 × 10^−30^
	rs1800562	HFE (C282Y)	A	G	0.067	1446	−0.479	0.016	8.90 × 10^−196^
	rs855791	TMPRSS6 (V736A)	A	G	0.446	47	−0.055	0.007	1.38 × 10^−14^
Transferrin saturation	rs1799945	HFE (H63D)	C	G	0.85	162	−0.231	0.01	5.13 × 10^−109^
	rs1800562	HFE (C282Y)	A	G	0.067	2126	−0.479	0.016	2.19 × 10^−270^
	rs855791	TMPRSS6 (V736A)	A	G	0.446	889	−0.19	0.008	6.41 × 10^−137^

### 2.3. Outcome data

GWAS statistics related CHD and related CVD can be extracted from the corresponding authoritative consortium or cohort studies.

Data for both HP and HF were obtained from UKBiobank, with a final sample of 462,933 people of European descent for HP (119,731 HP cases and 343,202 non-cases), and a final sample of 361,194 people of European descent for HF (1,405 HF cases and 359,789 non-cases) (19). GWAS resources for coronary atherosclerosis are based on data from the UKBiobank consortium for a total of 361,194 individuals of European descent including 14,334 AS patients and 346,860 healthy controls (20), and FinnGen for a total of 211,203 individuals of European descent including 23,363 AS patients and 187,840 healthy controls. The second is based on FinnGen data for 211,203 individuals of European descent, including 23,363 AS patients and 187,840 healthy controls, available on the FinnGen study website (https://finngen.gitbook.io/documentation/) (20, 21). CHD Statistical data were obtained from the coronary artery genome-wide replication and meta-analysis (CARDIoGRAM) and coronary artery disease genetics (CARDIoGRAMplusC4D) GWASmeta-analysis, which included 60,801 cases and 123,504 controls (22). Statistics for MI were also obtained from the Coronary Artery Whole Genome Replication and Meta-Analysis (CARDIoGRAM) and Coronary Artery Disease Genetics (CARDIoGRAMplusC4D) GWASmeta-analysis, which included 43,678 cases and 128,199 controls (22), and FinnGen which included 12,801 cases and 187,840 controls (21). We used aggregated data from published GWAS that referenced the original definitions of the diseases in their GWAS without any modifications. The specific data sources used are in Supplementary Table S4.

### 2.4. Statistical analysis

MR analysis was performed using five methods, inverse variance weighting (IVW) under a multiplicative random effects model, MR-Egger, Weighted median, Simple mode, and Weighted mode. IVW was performed by combining the Wald ratio estimates for each individual SNP will be one causal estimate for each risk factor (23). A sensitivity analysis is required to test the validity and robustness of the IVW estimates due to invalid instrumental bias and polymorphism.

Sensitivity analyses include heterogeneity tests, genetic pleiotropy tests, and the “leave-one-out” method (19). In sensitivity analysis, we can use the weighted median method to check for invalid instrumental bias to estimate the multiplicity of potential causal effects or the inclusion of invalid instruments (24), while the use of MR-Egger regression can explain both the dilution bias of the skewed regression, with the mean level of multiplicity consisting of the intercept term (24, 25). Subsequently, symmetries can be visualized using funnel plots, and if they are skewed in one direction, they indicate a potential multiplicative effect (26). Cochran's Q test was also used to estimate the heterogeneity between the Wald ratio estimated for the different SNPs (27). Finally, to identify all genetic variants potentially affecting SNPs, we performed a “leave-one-out” analysis, whose fluctuations in results before and after removal of SNPs may reflect unstable associations.

To further investigate the relationship between iron status biomarkers and CHD and related CVD, we separately selected the iron status biomarkers with positive MR analysis results as described above and each SNP of the corresponding disease for two-sample MR analysis again, respectively, to obtain a more accurate estimate of the causal effect of each iron status biomarker and disease. The characteristics and summary data of the separately selected SNPs are shown in Supplementary Table S2.

“Two SampleMR” (version 0.5.6) of R software (version 4.2.1) was used for all analyses. *P* values less than 0.05 were considered statistically significant.

## 3. Result

Genetically determined higher serum iron was negatively associated with higher odds of AS (OR = 0.995, 95% CI = 0.992–0.998, *p* = 0.002). The same results were obtained again using each SNP (OR = 0.996,95% CI = 0.992–0.998, *p* = 0.0009). Higher TS was negatively associated with higher odds of MI(Finn) (OR = 0.885, 95% CI = 0.797–0.982, *p* = 0.02). A repeat MR analysis using each SNP yielded no causal relationship between TS and MI (Finn) (*p* = 0.657) while higher TS was negatively associated with higher odds of MI (OR = 0.939, 95% CI = 0.886–0,996, *p* = 0.037), using the inverse variance-weighted approach. Moreover, the sensitivity analysis revealed that the selected instruments did not differ horizontally (*p*-values >0.05 for MR-Egger intercepts) or heterogeneously (*p*-values >0.05 for Cochran's Q statistic). In addition, MR-Egger regression and funnel plot appearance analyses showed a poor possibility of horizontal polymorphism (all *p*-values for MR-Egger intercept > 0.05) and visually, the leave-one-out analysis plot proved that the results were not altered by the removal of any SNPs and the results remained quite robust. The remaining MR analyses were negative that iron status markers were not causally associated with HP, CHD or HF.

**Figure 2:**
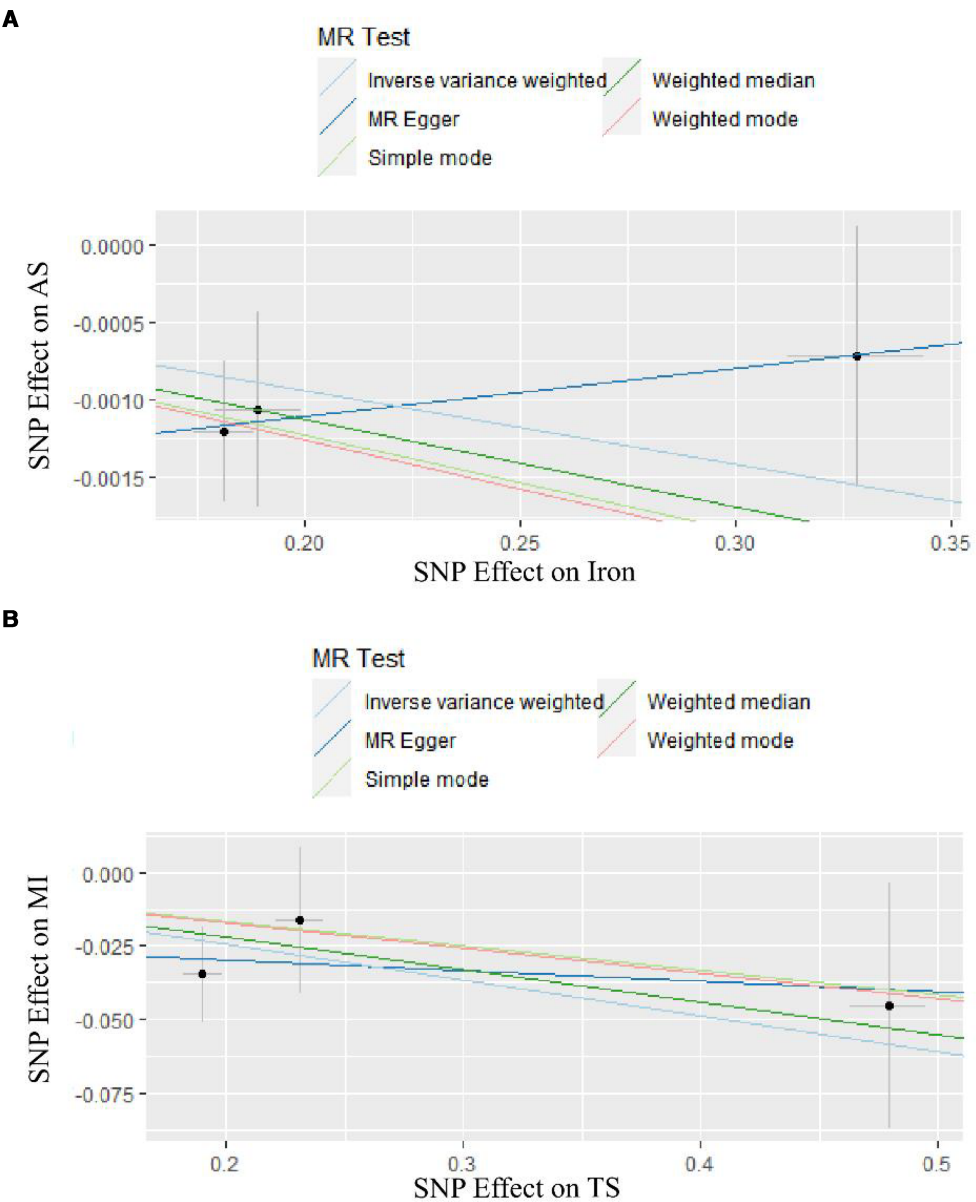
The result of two-sample MR analyses. (**A**) is the result of two samples MR analyses of Iron. (**B**) is the result of two samples MR analyses of TS.

**Figure 3:**
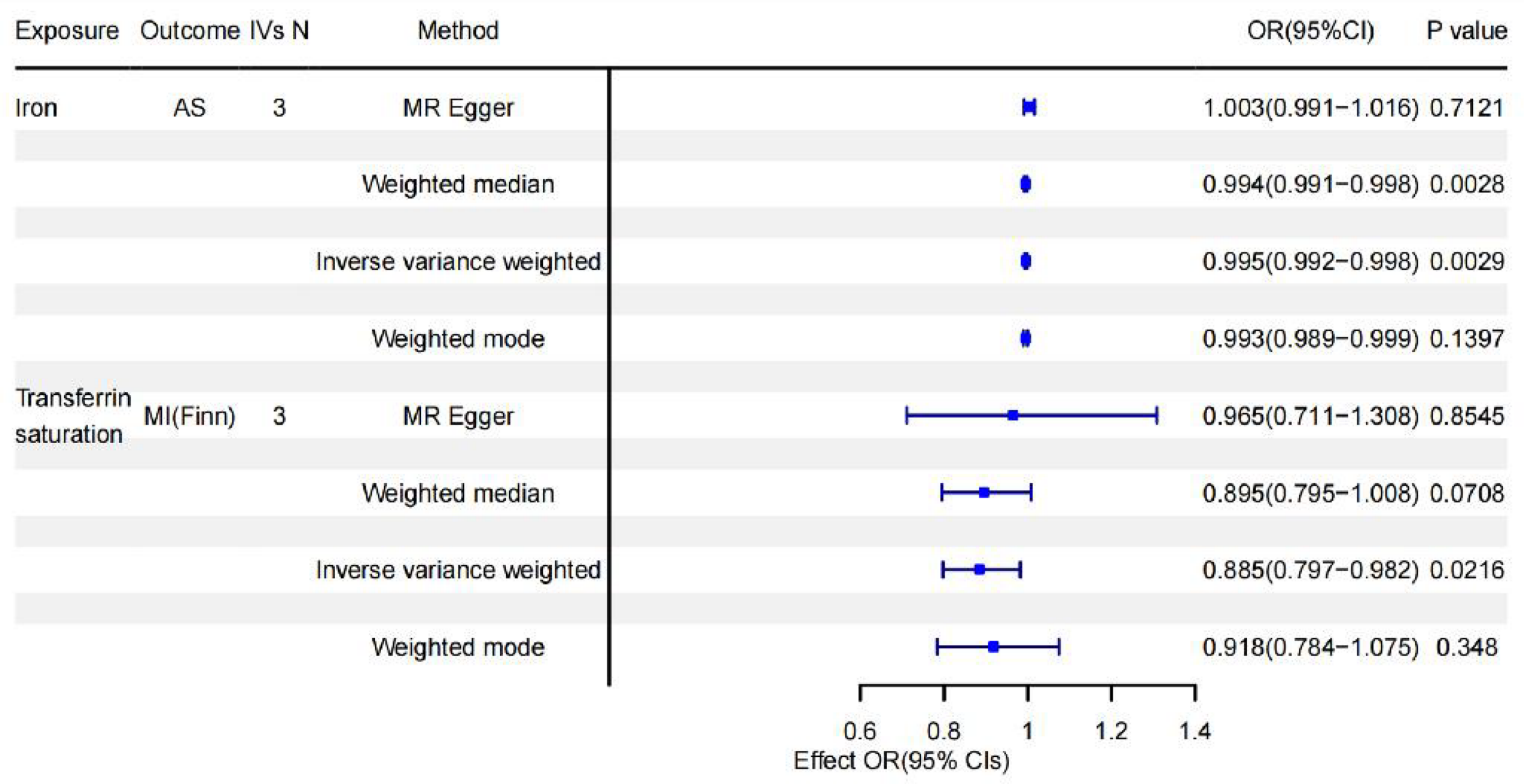
Forest plot summarizing causality of positive results.

**Figure 4:**
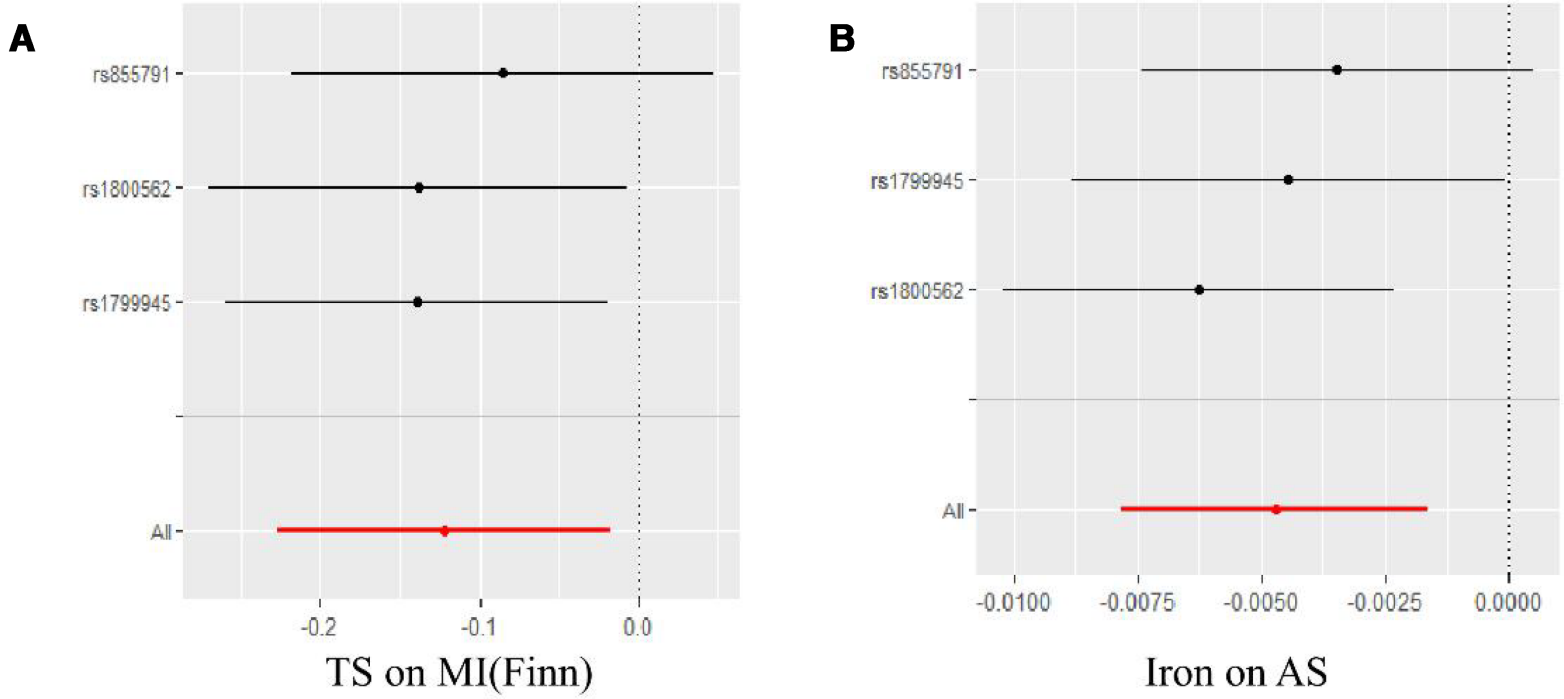
Leave-one-outanalyses of positive results in the causal relationship between iron status and CHD and related CVD. (**A**) is the result of TS and MI(Finn), and (**B**) is the result of Iron and AS.

Complete results are presented in Supplementary Figure S2. And the positive results were presented in Figure 2. The IVW results are shown in Table 2. Results of MR Analysis are expressed as the ORs for a positive result per one standard deviation (SD) increase for each iron biomarker, as shown in Figure 3 and Supplementary Figure S4. The leave-one-out analysis plot proves that removing any SNP does not change the results and is quite robust. The leave-one-out analysis graph of positive results are presented in Figure 4, and all results are presented in Supplementary Figure S3. The results of MR Analysis of AS and MI with the separately selected SNPs associated with Iron and TS are presented in Supplementary Table S3 and Supplementary Figure S3. The results of the positive results reanalysis are expressed as ORs per one standard deviation (SD) increase in positive results for each iron biomarker, as shown Supplementary Figure S4.

**Table 2:** The results of IVW.

		HP			AS			AS(Finn)			CAD		
Exposure	nSNP	Beta	SE	*P*	Beta	SE	*P*	Beta	SE	*P*	Beta	SE	*P*
Iron	3	0.028	0.0194	0.147	−0.005	0.0016	0.002	−0.031	0.0805	0.704	−0.075	0.0461	0.104
Ferritin	3	0.064	0.0402	0.110	−0.008	0.0053	0.116	0.003	0.1998	0.989	−0.189	0.1008	0.060
Transferrin	3	−0.032	0.0182	0.081	0.001	0.0036	0.726	−0.105	0.0687	0.127	0.042	0.0791	0.595
Transferrin saturation	3	−0.001	0.0221	0.959	−0.002	0.0025	0.446	−0.071	0.0474	0.134	0.006	0.0579	0.920

		MI(Finn)			MI			HF		
Exposure	nSNP	Beta	SE	*P*	Beta	SE	*P*	Beta	SE	*P*
Iron	3	−0.082	0.0958	0.390	−0.062	0.0426	0.145	−1.43 × 10^−4^	0.0005	0.779
Ferritin	3	−0.084	0.2624	0.746	−0.176	0.0988	0.074	−4.66 × 10^−4^	0.0011	0.676
Transferrin	3	−0.101	0.1160	0.382	0.063	0.0593	0.287	4.20 × 10^−4^	0.0005	0.429
Transferrin saturation	3	−0.122	0.0532	0.022	0.021	0.0484	0.668	1.16 × 10^−4^	0.0004	0.776

## 4. Discussion

We applied MR to analyze the causal relationship between four biomarkers of iron status and CHD and related CVD and concluded that there is a partial causal relationship between systemic iron status and CHD and related CVD. In this MR analysis, there was a negative correlation between serum iron levels and coronary AS and between TS and MI. The same conclusion was reached when the MR analysis was repeated for AS and MI using the individual SNPs for serum iron and TS, respectively. It implies that a genetically determined increase in serum iron decreases the risk of AS and a genetically determined increase in TS decreases the risk of MI. Based on the fact that TS is one of the biomarkers reflecting systemic iron status, we can speculate that genetically determined ID may lead to an increased risk of CHD.

In a study of patients on hemodialysis, it was found that high doses of intravenous iron reduced the risk of MI compared to lower doses (28). More noteworthy is the finding in another study that patients who were iron deficient at the time of acute coronary syndrome had a significantly higher risk of cardiovascular mortality and nonfatal myocardial infarction over 4 years (6), results that are consistent with those obtained in this paper. However, in patients with st-segment elevation myocardial infarction treated with percutaneous coronary intervention, patients with ID were found to have a better in-hospital prognosis. The investigators speculate that this may be related to the ability of ID to reduce myocardial ischemia-reperfusion injury (29). Based on the above, we hypothesize that the effect of iron on MI is not only that higher iron levels reduce the risk of MI, but also that ID increases the long-term effects of a worse incidence of MI. Considering the small causal effect, these MR-based analyses should be referenced with caution. Previous MR analyses on iron status and carotid plaque have also shown that genetically determined iron levels increase carotid plaque with a protective effect (30). This laterally supports the results of the MR of serum iron with AS in this paper. However, some studies have yielded contradictory results, suggesting that high iron promotes the progression of atherosclerosis and increases its severity (31). This discrepancy may stem from the fact that the occurrence of AS is influenced by genetic and environmental factors, and we analyzed the relationship from a genetic perspective, focusing on lifetime effects rather than short-term effects.

Some of these results contradict the results of the reported papers, which may be explained by the fact that no correction for disease subtyping population stratification and population pedigree was performed when processing GWAS data for CHD, in which cases of acute coronary syndrome, coronary artery bypass grafting, and percutaneous coronary revascularization were included in the CHD cases used (17). As part of the current MR study, three SNPs were selected from a recent large-scale GWAS. Among them, mutations in the gene HFE include rs1800562 (also known as C282Y) and rs1799945 (also known as H63D). The missense mutation in rs1800562 retains the dysfunctional HFE in the endoplasmic reticulum instead of being transported to the plasma membrane, resulting in the parenchymal iron overload disease of hereditary hemochromatosis (HH) (32). A recent study of 2890 European patients with C282Y-pure HH found a significantly lower risk of cardiovascular disease in C282Y-pure HH patients compared to age-matched subjects (33). Moreover, compared to HFE wild-type study participants, C282Y-positive participants had lower total cholesterol and LDL-C levels (34). In addition, a large GWAS study showed that genetic variants on H63D are associated with the prevalence of hypertension (35). It has been hypothesized that H63D causes toxic damage to the vascular endothelium by increasing iron stores and producing oxidative stress (36). However, the exact mechanism by which H63D causes hypertension remains unclear. Hepcidin, a key role of iron homeostasis, is a peptide hormone synthesized in hepatocytes that regulates cellular iron output by interacting with iron transport proteins (37, 38). Mutations in TMPRSS6 result in excessive iron uptake by encoding a type II plasma membrane serine protease, called matriptase-2, which inhibits hepcidin (39). Moreover, rs1800562 and rs1799945 have been reported to affect hepcidin (40). In conclusion, the above indicates that all three SNPs above can be involved in the occurrence and development of CVD by regulating iron metabolism.

Iron, one of the most essential nutrients, has been shown in many previous studies to be associated with abnormal iron status and CVD. In a cohort of 12,164 individuals from three European populations ID was associated with a 24% increased risk of CHD, a 26% increased risk of CVD death and a 12% increased risk of all-cause mortality, with 5.4% of deaths, 11.7% of CVD deaths and 10.7% of CHD events attributable to iron deficiency(ID) (41). ID is also an observable indicator of HF, about half of HF patients having ID according to the definition of ID (42, 43), with a specific prevalence of ID in chronic HF of about 47%–68%, and the more severe the HF, the more likely it is to occur (44). A double-blind randomized trial showed that intravenous iron carboxymaltose supplementation reduced the risk of HF hospitalization in iron-deficient patients with stable left ventricular ejection fraction below 50% after an acute HF episode, but had no significant effect on their risk of cardiovascular death (45). However, although observational studies and MR analyses have indicated that ID increases the risk of CHD, there are no relevant experimental results indicating that improving ID reduces the risk of developing CHD. In an epic-Heidelberg study,serum ferritin concentrations were associated with IM risk and cardiovascular disease mortality, but were not statistically significant after adjustment (46). Thus the effect of iron deficiency on CHD and CVD remains questionable. Therefore, the hypothesis has been put forward that the effect of iron on the heart lies in the increased oxidative stress due to iron overload (47).

Ferroptosis, in which the key factors of iron toxicity are Fe^2+^ accumulation and lipid peroxidation, is a novel form of cell death with unique genetic, biochemical, morphological and metabolic characteristics in contrast to apoptosis, necroptosis and scorch death (13). As iron plays a key role in catalyzing phospholipid peroxidation in ferroptosis, unrestricted lipid peroxidation is exactly one of the hallmark symptoms of ferroptosis (48) Lipid peroxidation is subject to molecular oxidation reactions that generate peroxyl radicals, and if not eventually reduced to the corresponding alcohols, the propagation of the radical-mediated reactions leads to the formation of numerous secondary products that disrupt cell membrane integrity and eventually lead to cell death (49). On the other hand, excessive intracellular iron accumulation is associated with an overproduction of ROS, which leads to extensive oxidation of polyunsaturated fatty acids and disruption of cell membrane structure, ultimately leading to cell death (50). Iron overload has also been suggested as one of the potential mechanisms of myocardial I/R injury (51). An increase in Fe^2+^ concentration in cardiomyocytes has been reported to be observed in I/R-treated rats (52, 53). The upregulation of TfR1 in I/R-treated rat hearts was associated with elevated iron content, and the inhibiting of TfR1 expression accompanied a decrease in iron content and reduced I/R injury, so hypoxia may be responsible for causing I/R iron overload (54). Consistently, iron death is observed during atherosclerosis. The likelihood of atherosclerosis can be reduced by inhibiting iron death in aortic endothelial cells to attenuate lipid peroxidation and endothelial dysfunction (55).

Morever, it has been shown that systemic iron satuts are not equal to cardiac iron levels, so systemic iron disorders do not directly affect cardiac iron satuts (56, 57), and further studies are needed to determine whether systemic iron supplements have a beneficial effect on CVD. Studies have shown that a low-iron diet fed to rats results in reduced levels of iron transport proteins in the rat heart, resulting in reduced iron output from heart cells (58), suggesting that iron levels in the heart are not necessarily affected by a low-iron diet. Consistently, in another study of a mild cardiomyopathy model in FthMCK/MCK mice both showed reduced cardiac iron levels without changes in serum or skeletal muscle iron levels, but after 4 weeks on a high iron diet, cardiac GSH levels in mice were reduced due to increased cardiac iron levels, resulting in cardiomyocyte ferroptosis.

Herein, the decrease in serum iron does not correlate positively with the decrease in cardiac iron levels,which cellular iron levels may be increased and be caused ferroptosis in cardiomyocytes while iron supplementation (59). Combined with the results of the Mendelian study in this paper, we can propose the hypothesis that the effect of decreased systemic iron status on CVD lies in overall cardiomyocyte function. Systemic iron status is negatively correlated with diseasewhen there are more normal cardiomyocytes than damaged cardiomyocytes in a cardiovascular disease event, whereas systemic iron status is not correlated with disease development when the situation is reversed. It is possible that iron supplementation at this point will lead to cellular iron overload, which will promote the development of cellular ferroptosis as a result. Therefore, considering the potentially deleterious effects of ID and iron overload, iron status intervention strategies may not be beneficial for patients in the CHD and related CVD without ID. In conclusion, based on the effect of systemic iron status on cardiac cellular iron levels, future studies should aim to identify CHD and related CVD phenotypes that would benefit from improved ID and to investigate their specific pathophysiological mechanisms.

The study we conducted has several limitations. First, because individual data were not available and we only performed summary statistics, the CHD and related CVD data used in this study were not stratified by disease subtype, such as dividing CHD into stable angina pectoris and acute non-ST-segment elevation myocardial infarction. Indeed, iron status markers may have a stronger association with specific subtypes of CHD or at acute onset. Therefore, further studies are needed to investigate whether similar results exist in patients of different races, different subtypes of CHD and CVD, and different degrees of disease severity. Second, for some exposures, the body may have mechanisms to respond to this exposure level, such as systemic iron status that is not synchronized with cellular iron levels, which hinders our study of iron metabolism and CHD and related CVD from macroscopic regulators to microscopic pathophysiological changes in the present study. Considering the small causal effect of this analysis, the MR Analysis estimates of this study should be interpreted with caution, and the inferences and assumptions in this paper should be referred to with caution. Nevertheless, the present study provides some clues to the pathophysiology and therapeutic exploration of CHD and related CVD. We expect future studies to delve into the relationship between CHD and related CVD and iron status, which may provide new insights into the prevention and treatment of CHD.

## Data Availability

The original contributions presented in the study are included in the article/**Supplementary Material**, further inquiries can be directed to the corresponding authors.
