# Supplementary material for: Mendelian randomization study reveals a causal relationship between serum iron status and coronary heart disease and related cardiovascular diseases
Source: Front Cardiovasc Med. 2023 Jun 13;10:1152201. doi: 10.3389/fcvm.2023.1152201 (PMC10294586; doi:10.3389/fcvm.2023.1152201)
Supplement: Supplementary file 1 [file Datasheet1.pdf]

Supplementary Fig.1

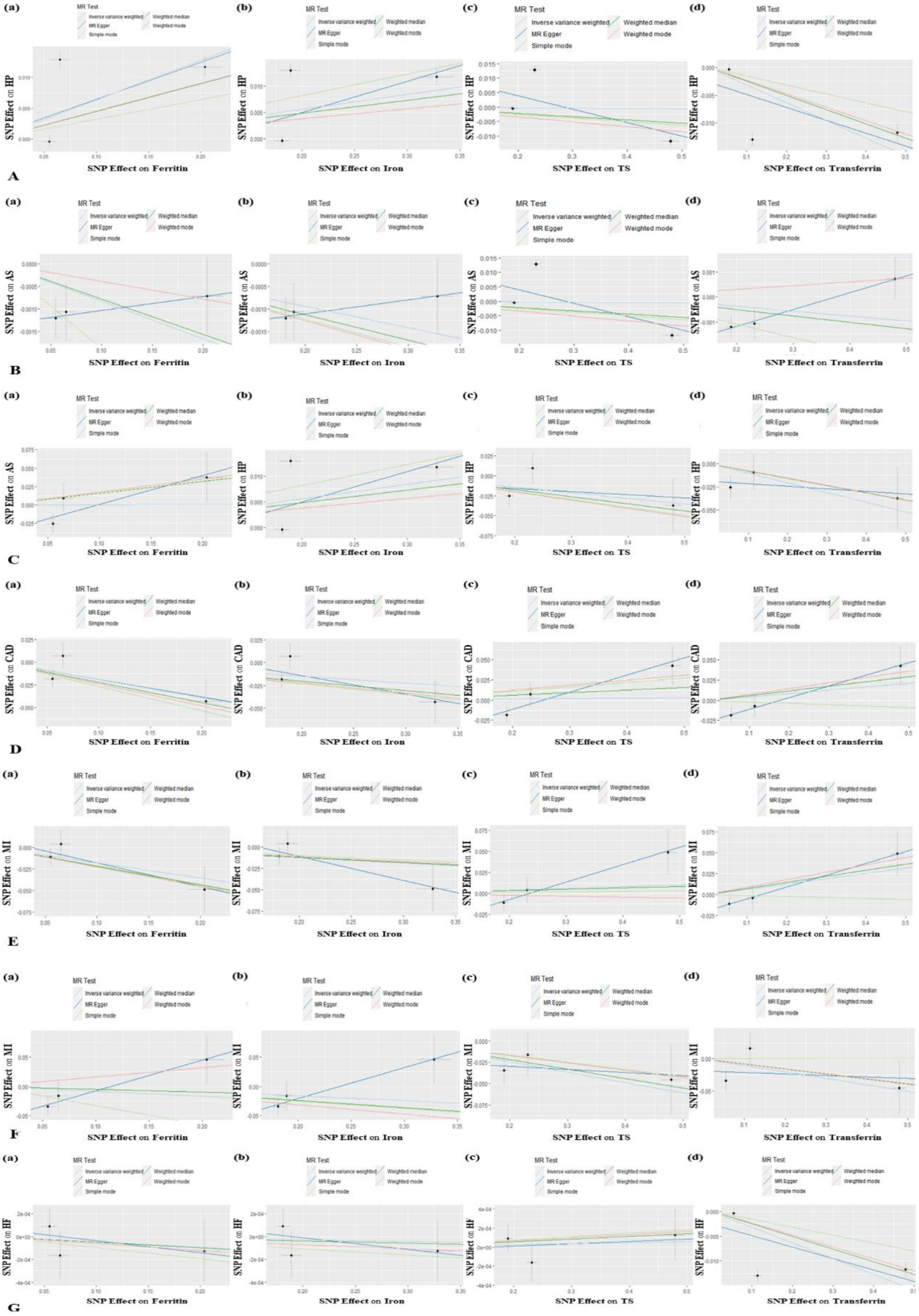

Supplementary Fig.1 The complete results of the Five MR analysis methods

Supplementary Fig. 2

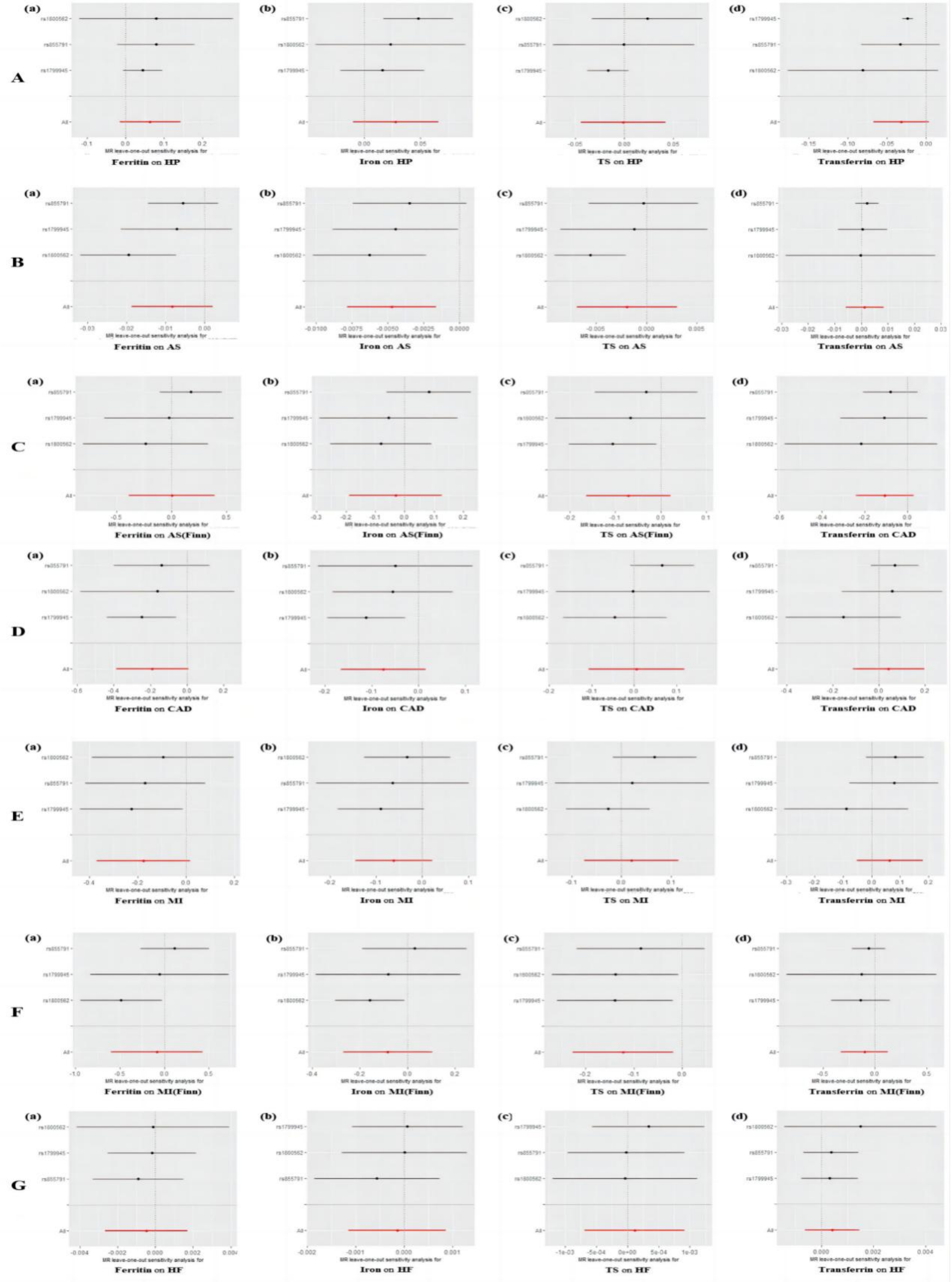

Supplementary Fig.2 The leave-one-out analysis of the causal relationship between iron status and CHD and related CVD

Supplementary Fig. 3

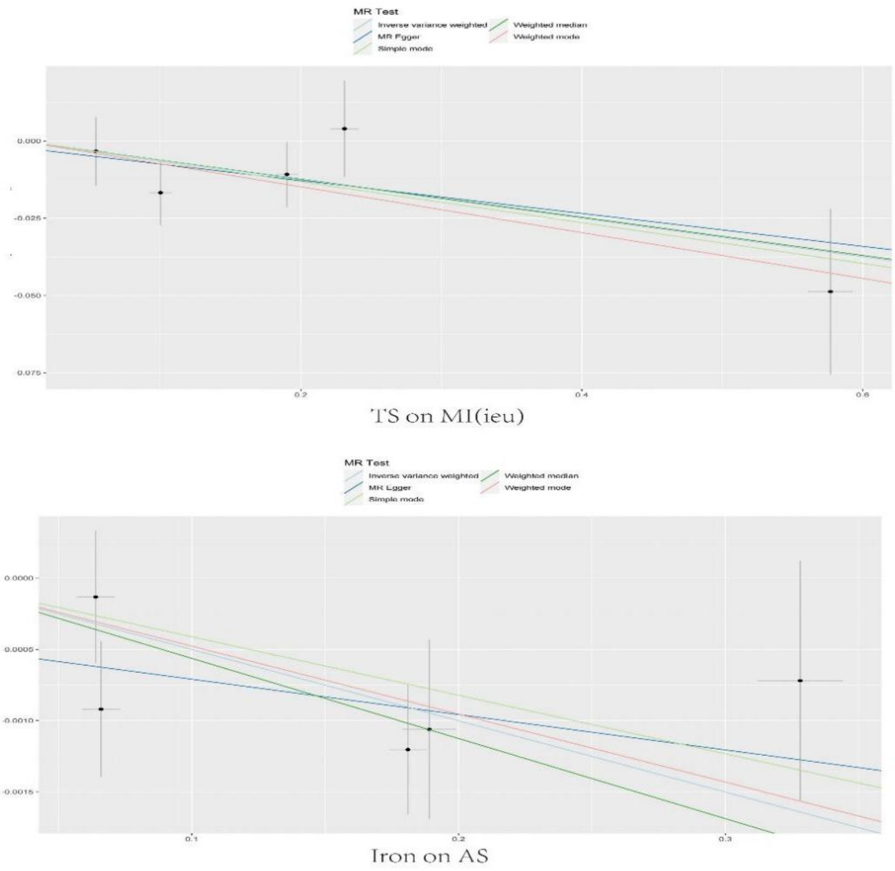

Supplementary Fig. 3 The result MRanalysis of the separately selected SNPs of iron status biomarker of positive results in Mr Analysis and the corresponding CHD and related vascular diseases

Supplementary Fig. 4

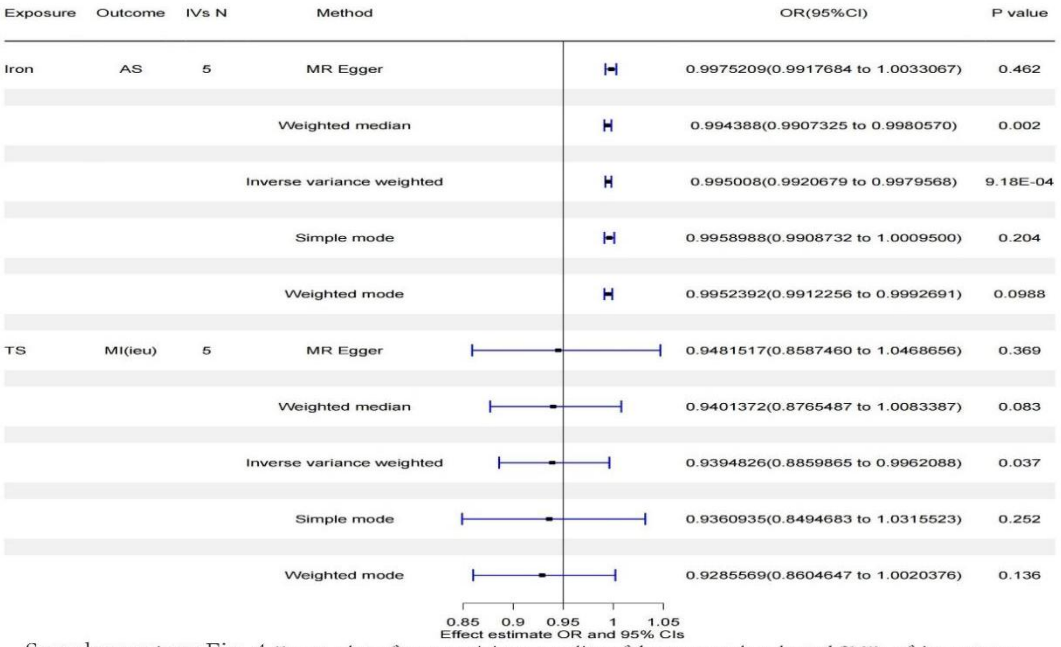

Supplementary Fig. 4 Forest plot of summarizing causality of the separately selected SNPs of iron status biomarker of positive results in Mr Analysis and the corresponding CHD and related vascular diseases

Supplementary Fig. 5

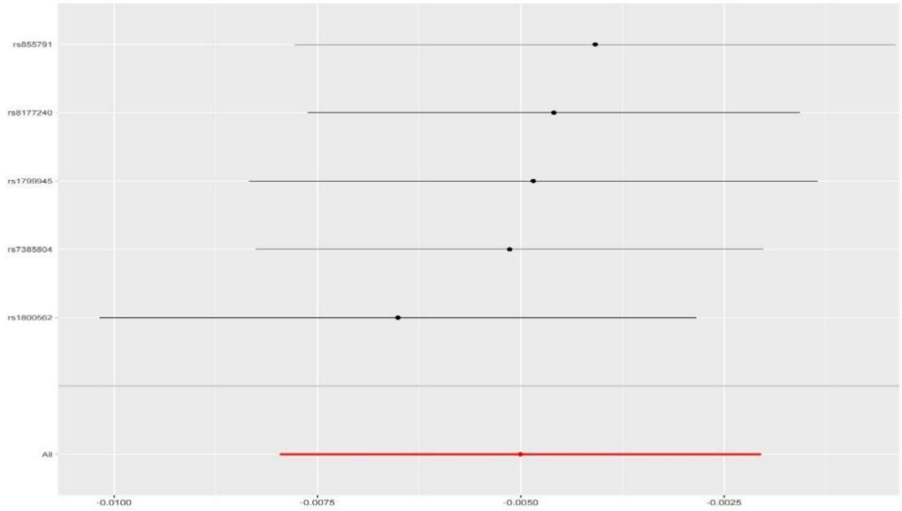

TS on MI(ieu)

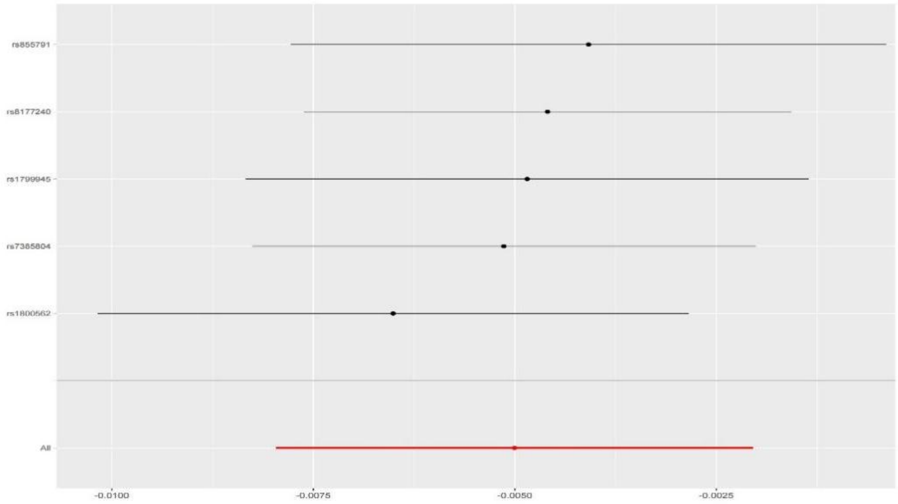

Iron on AS

Supplementary Fig. 5 Leave-one-out analysis of the separately selected SNPs of iron status biomarker of positive results in Mr Analysis and the corresponding CHD and related vascular diseases

Supplementary Table 1. The characteristics of SNPs

CHR	SNP	Position	Nearest Gene	EA	OA	eaf	Phenotype	beta	se	p	F
2	rs744653	190,378,750	WDR75(SLC40A1)	T	C	0.854	Iron	0.004	0.01	0.702	0.195
							Transferrin	0.068	0.01	1.35E−11	56.531
							TS	−0.028	0.011	0.0084	9.575
							Ferritin	−0.089	0.01	8.37E−19	96.919
3	rs8177240	133,477,701	TF	T	G	0.669	Iron	−0.066	0.007	6.65E−20	94.654
							TS	−0.380	0.007	3.29E−615	31.337
							Saturation	0.1	0.008	7.24E−38	217.842
							Ferritin	0.021	0.007	0.0039	9.566
3	rs9990333	195,827,205	TFRC	T	C	0.46	Iron	0.017	0.007	0.014	7.031
							Transferrin	−0.051	0.007	1.95E−13	63.359
							TS	0.039	0.007	7.28E−8	37.031
							Ferritin	0.001	0.007	0.878	0.024
6	rs1800562	26,093,141	HFE	A	G	0.067	Iron	0.328	0.016	2.72E−97	667.644
							Transferrin	−0.479	0.016	8.90E−196	1446.197
							TS	0.577	0.016	2.19E−270	2126.826
							Ferritin	0.204	0.016	1.54E−38	256.119
6	rs1799945	26,091,179	*HFE* (H63D)	C	G	0.85	Iron	−0.189	0.01	1.10E−81	450.161
							Transferrin	0.114	0.01	9.36E−30	162.825
							TS	−0.231	0.01	5.13E−109	675.529
							Ferritin	−0.065	0.01	1.71E−10	52.816
7	rs7385804	100,235,970	TFR2	A	C	0.621	Iron	0.064	0.007	1.36E−18	94.599
							Transferrin	−0.003	0.007	0.728	0.207
							TS	0.054	0.008	6.07E−12	67.309
							Ferritin	0.015	0.007	0.039	5.187
8	rs4921915	18,272,466	NAT2	A	G	0.782	Iron	0.004	0.009	0.633	0.267
							Transferrin	0.079	0.009	7.05E−19	104.424
							TS	−0.026	0.009	0.0036	11.289
							Ferritin	0.001	0.009	0.886	0.017
9	rs651007	136,153,875	ABO	T	C	0.202	Iron	−0.004	0.009	0.611	0.253
							Transferrin	−0.001	0.009	0.916	0.0158
							TS	−0.006	0.009	0.498	0.568
							Ferritin	−0.050	0.009	1.31E−8	39.501
11	rs6486121	13,355,770	ARNTL	T	C	0.631	Iron	−0.009	0.007	0.202	1.847
							Transferrin	−0.046	0.007	3.89E−10	48.301
							TS	0.015	0.008	0.048	5.131
							Ferritin	0.006	0.007	0.424	0.821
11	rs174577	61,604,814	FADS2	A	C	0.33	Iron	0.001	0.007	0.878	0.022
							Transferrin	0.062	0.007	2.28E−17	83.382
							TS	−0.025	0.008	0.0016	13.538
							Ferritin	−0.012	0.007	0.098	3.118

17	rs411988	56,709,034	TEX14	A	G		Iron	−0.002	0.007	0.77	-99599.011
						0.564	Transferrin	0.014	0.007	0.052	4.721
							TS	−0.012	0.007	0.115	3.468
							Ferritin	−0.044	0.007	1.59 E−10	46.67
22	rs855791	37,462,936	TMPRSS6	A	G		Iron	−0.181	0.007	1.32E−139	805.843
						0.446	Transferrin	0.044	0.007	1.98E−9	46.895
							Saturation	−0.190	0.008	6.41E−137	889.466
							Ferritin	−0.055	0.007	1.38E−14	73.313

CHR—chromosome;EA—effect alleles;OA—other alleles;EAF—effect alleles frequency; SE—standard error;TS—transferrin saturation

Supplementary Table 2. The characteristics and summary data of the separately selected SNPs associated with Iron and TS

SNP	Position	CHR	Nearest Gene	EA	OA	eaf	beta	se	p	F
**Iron**										
rs1800562	26,093,141	6	HFE (C282Y)	A	G	0.067	0.328	0.016	2.72E−97	668
rs1799945	26,091,179	6	HFE (H63D)	C	G	0.85	−0.189	0.01	1.10E−81	450
rs855791	37,462,936	22	TMPRSS6 (V736A)	A	G	0.446	−0.181	0.007	1.32E−139	806
rs8177240	133,477,701	3	TF	T	G	0.669	−0.066	0.007	6.65E−20	95
rs7385804	100,235,970	7	TFR2	A	C	0.621	0.064	0.007	1.36E−18	95
**TS**										
rs1800562	26,093,141	6	HFE (C282Y)	A	G	0.067	0.577	0.016	2.19E−270	2127
rs1799945	26,091,179	6	HFE (H63D)	C	G	0.85	−0.231	0.01	5.13E−109	676
rs855791	37,462,936	22	TMPRSS6 (V736A)	A	G	0.446	−0.19	0.008	6.41E−137	889
rs8177240	133,477,701	3	TF	T	G	0.669	0.1	0.008	7.24E−38	218
rs7385804	100,235,970	7	TFR2	A	C	0.621	0.054	0.008	6.07E−12	67

Supplementary Table 3. The results of MR Analysis of AS and MI with the separately selected SNPs associated with Iron and TS

	**N**	**Methods**	**AS**			**AS(Finn）**		
			**Beta**	**SE**	**p**	**Beta**	**SE**	**p**
**Iron**	5	MR Egger	-0.0025	0.003	0.462	-0.0287	0.1348	0.845
		Weighted median	-0.0056	0.0019	0.002	-0.0213	0.0646	0.742
		Inverse variance weighted	-0.005	0.0015	0.0009	-0.019	0.0588	0.746
		Simple mode	-0.0041	0.0027	0.0027	0.0813	0.1177	0.528
		Weighted mode	-0.0048	-0.0048	0.099	-0.1248	0.079	0.189

	**N**	**Methods**	**MI**			**MI(Finn)**		
			**Beta**	**SE**	**p**	**Beta**	**SE**	**p**
**TS**	5	MR Egger	-0.0532	0.0505	0.369	-0.0056	0.1105	0.963
		Weighted median	-0.0617	0.0356	0.083	0.0348	0.0646	0.59
		Inverse variance weighted	-0.0624	0.0299	0.037	-0.0258	0.0582	0.657
		Simple mode	-0.066	0.0494	0.252	0.0706	0.114	0.569
		Weighted mode	-0.0741	0.0398	0.136	0.0550	0.0982	0.605

Supplementary Table 4. Specific data sources included in the study

**Phenotype**	**Consortium**	**Sample Size**	**N Cases**	**N Controls**	**Population**	**Phenotype descriptions**	**MRC-IEU id:**
Serum iron status	GIS Consortium	48972	NA	NA	Europeans	Data from 11 discovery and 8 replication cohorts were used in the meta-analysis, which combined data from 48972 European subjects	NA
HF	Neale Lab	361194	1405	359789	European	Data included 361,194 males and females of European ancestry, including 1405 HF cases and 359,789 controls	ukb-d-HEARTFAIL
CAD	CARDIoGRAMplus4CD	184305	60801	123504	Mixed	Data include subjects with confirmed acute coronary syndromes, coronary artery bypass grafting, percutaneous coronary revascularization, chronic stable angina and stenosis of one or more coronary vessels greater than 50% pectoris, in populations including European, East Asian, South Asian,Hispanic,and African American populations	ieu-a-7
MI	CARDIoGRAMplus4CD	171875	43676	128199	Mixed	Data population sources include 171,875 males and females of European, East Asian, South Asian, Hispanic,and African American origin, with 43,676 MI cases and 128,199 controls.	ieu-a-798
MI	FinnGen Release 3	124008	8234	115774	European	Data included 124,008 males and females of European ancestry, including 8,234 MI cases and 115,774 controls	finn-b-I9_MI

AS	Neale Lab	361194	14334	346860	European	Data included 361,194 males and females of European ancestry, including 14,334 AS cases and 346,860 controls	ukb-d-I9_CORATHER
AS	FinnGen Release 3	130746	14972	115774	European	Data included 130,746 males and females of European ancestry, including 14,972 AS cases and 115,774 controls	finn-b-I9_CORATHER
HP	MRC-IEU	462933	119731	343202	European	Data on self-reported hypertension from UK Biobank included 462,933 males and females of European ancestry, including 199 ,731 HP cases and 343,202 controls	ukb-b-14057
